# Maternal hypothyroidism and subsequent metabolic outcomes in children: a systematic review and meta-analysis

**DOI:** 10.1186/s12887-024-04963-0

**Published:** 2024-08-01

**Authors:** Lucy Zhao, Inthuja Selvaratnam, Jessie Cunningham, Kristian B. Filion, Sonia M. Grandi

**Affiliations:** 1 Faculty of Health Sciences, McMaster University, Hamilton, ON Canada; 2 Department of Child Health Evaluative Sciences, The Hospital for Sick Children, Peter Gilgan Centre for Research and Learning, Toronto, ON Canada; 3 Centre for Clinical Epidemiology, Lady Davis Institute, Jewish General Hospital, Montreal, QC Canada; 4 Department of Epidemiology, Biostatistics and Occupational Health, McGill University, Montreal, Canada; 5 Department of Medicine, McGill University, Montreal, QC Canada; 6 Division of Epidemiology, Dalla Lana School of Public Health, University of Toronto, Toronto, ON Canada

**Keywords:** Hypothyroidism, Pregnancy, Metabolic outcomes, Offspring, Systematic review

## Abstract

**Introduction:**

As the fetus relies on maternal thyroid hormones in early pregnancy, maternal hypothyroidism plays an important role in fetal development. However, the association between maternal hypothyroidism and metabolic disease in offspring is unclear.

**Objective:**

To examine the association between maternal hypothyroidism in pregnancy and metabolic outcomes (obesity, hypertension, type 2 diabetes mellitus, and dyslipidemia) in children < 18 years.

**Methods:**

We systematically searched 5 databases from inception to May 2023. Eligible studies included cohort, case-control, and randomized controlled trials involving children born to mothers with or without hypothyroidism in pregnancy. Data were pooled across studies using random-effects models for outcomes reported in at least three studies. Quality assessment was performed using the ROBINS-E tool for observational studies and the Cochrane Risk of Bias tool for trials.

**Results:**

The search identified 3221 articles, of which 7 studies were included (1 trial, 6 observational). All studies were conducted outside of North America and ranged in size from 250 to > 1 million children. The follow-up time ranged from 6 to 20 years. Included studies support an increased risk of hypertension and glucose dysregulation in offspring exposed to maternal hypothyroidism (hypertension: OR 1.08, 95% CI 0.75, 1.57 and HR 1.81, 95% CI 1.21, 2.69; diabetes: RR 2.7, 95% CI 0.7, 10). In the pooled analysis, maternal hypothyroidism was not associated with obesity in offspring (OR 1.04, 95% CI 0.64, 1.70).

**Conclusion:**

This study found inconsistent evidence on the association between maternal hypothyroidism in pregnancy and metabolic outcomes in offspring, though associations with hypertension and glucose dysregulation are possible.

**Supplementary Information:**

The online version contains supplementary material available at 10.1186/s12887-024-04963-0.

## Introduction

Hypothyroidism occurs in approximately 2–3% of pregnant women, with < 1% of women presenting with overt hypothyroidism and 2-2.5% with subclinical hypothyroidism (SCH) [1, 2]. Overt hypothyroidism is characterized by increased thyroid stimulating hormone (TSH) levels and decreased thyroxine (T4) concentrations, while SCH is characterized only by abnormal TSH levels [3]. Women with hypothyroidism may be asymptomatic or the symptoms may be masked by the hypermetabolic state of pregnancy making diagnosis and treatment for in pregnancy a challenge [3].

Untreated or inadequately managed hypothyroidism in pregnancy has been associated with maternal and fetal complications [2, 4]. Individuals with gestational hypothyroidism are at increased risk of maternal complications including anemia, gestational hypertension, and congestive heart failure, as well as fetal complications including miscarriage, stillbirth, perinatal death, low birth weight, and preterm delivery [1, 2, 5–7]. Children born to individuals with hypothyroidism in pregnancy have been found to be at greater risk of chronic disease development in childhood, such as cardiovascular disease, thyroid dysregulation and neurocognitive impairment [4, 8–10]. While physiological mechanisms have been explored to explain the role of maternal thyroid disease in offspring development, the influence of maternal hypothyroidism on the long-term risk of metabolic disease in children is less clear.

Thyroid hormones play an important role in regulating lipid and glucose metabolism as well as cardiovascular function, including cardiac contractility and output, blood pressure, and systemic vascular resistance [11, 12]. As the fetus is unable to produce its own thyroid hormone in early pregnancy, the fetus relies on maternal thyroid hormone supply until 12 to 14 weeks of gestation [2]. While maternal hypothyroidism has been linked to negative metabolic outcomes in the birthing parent, the role of thyroid dysfunction during fetal heart development and its influence on childhood metabolic risk is unclear. Research suggests that a disruption to the fetal supply of thyroid hormone during pregnancy can impair fetal thyroid function due to the physiological immaturity of the hypothalamic-pituitary thyroid axis, leading to subsequent abnormal fetal-placental glucose metabolism [13]. Exposure to maternal hypothyroidism in utero may also impact fetal organ development and gene expression through epigenetic modifications [10, 14]. Murine models have demonstrated that in utero exposure to maternal hypothyroidism influences cardiovascular and endocrine function in offspring through elevated blood pressure, altered renin-angiotensin system function, and increased glucose intolerance [15–17]. Observational studies suggest that maternal hypothyroidism may be associated with a greater risk of hypertension and diabetes mellitus (DM) in children, though the association with childhood obesity and other metabolic parameters remain unclear [10, 18–21]. Due to the heterogeneity of hypothyroidism definitions and outcome reporting in prior studies, a systematic review is needed to assess the existing evidence base.

To date, no systematic review has investigated the risk of metabolic outcomes in children following exposure to maternal hypothyroidism during pregnancy. Given the possible effect of maternal thyroid dysfunction on fetal development and metabolism, this systematic review and meta-analysis provides a synthesis of previous studies to assess the risk of metabolic outcomes in children exposed to maternal hypothyroidism during pregnancy.

## Methods

### Study design and registration

This systematic review was conducted according to a prespecified protocol and is reported following the reporting guidelines outlined in the Synthesis Without Meta-analysis (SWiM) and PRISMA (Preferred Reporting Items for Systematic Reviews and Meta-analyses) statements (Additional file 1). The protocol for this review was registered in the International Prospective Register of Systematic Reviews (PROSPERO; CRD42023426857).

### Search strategy

The search was developed by a professional medical librarian (JC) trained in knowledge synthesis (Additional file 2). The search was performed in the following bibliographic databases including MEDLINE, Embase, CINAHL, PubMed, and the Cochrane Library databases. The search strategy consisted of both controlled vocabulary, such as the National Library of Medicine’s MeSH (Medical Subject Headings), Emtree Subject Headings (Embase), CINAHL Subject Headings, and keywords. The databases were searched from inception to May 26, 2023, with no language restrictions. The search strategy was developed based on three main concepts including, children (sample population), maternal hypothyroidism during pregnancy (exposure), and metabolic outcomes in offspring (outcomes). The bibliographies of included studies were hand-searched to identify relevant studies that were not captured by our initial search. Independent manual searching was conducted in up to 5 pages in Google Scholar to identify additional citations that were not captured in the initial search.

### Study selection

Studies were eligible if they were conducted among children aged ≤ 18 years who were born to individuals who were diagnosed with hypothyroidism (subclinical or overt) prior to or during pregnancy. Eligible study designs included cohort studies, case-control studies, and randomized controlled trials conducted in humans and available in the English language. The exposure of interest in observational studies was maternal hypothyroidism. The intervention of interest in randomized controlled trials was levothyroxine, used to treat individuals with hypothyroidism in pregnancy. Cross-sectional studies, case reports, case series, and surveillance studies were excluded due to the absence of a control group and difficulty in establishing the temporality of exposure and outcomes in these studies. As results may not be finalized and subject to revisions, conference abstracts were excluded. Editorials, commentaries, policy statements, clinical guidelines, and book chapters were also excluded. Two independent reviewers (LZ, IS) conducted title, abstract, and full-text screening of identified articles using the Covidence software for systematic reviews (Veritas Health Innovation, Melbourne, Australia).

### Data extraction and outcomes

An electronic data extraction form was pilot tested on five randomly selected studies. Any changes were incorporated into the final data extraction form, as needed. Two independent reviewers (LZ, IS) performed data extraction. Data extracted from identified studies included study characteristics (year of publication, country, study design), population characteristics (maternal and offspring sociodemographic characteristics, median age at time of pregnancy, medications taken during pregnancy, comorbidities), and exposure status (method of ascertainment, data source, antibody and thyroid hormone levels, subtype of hypothyroidism, timing of hypothyroidism diagnosis). Data extracted on outcomes included definitions, absolute rates, age-standardized rates, and effect estimates and corresponding 95% confidence intervals (CI) for the following outcomes: obesity (dichotomous, categorical), body mass index (BMI) (continuous), waist circumference (WC) (dichotomous, continuous), hypertension (dichotomous), systolic blood pressure (SBP) (continuous), diastolic blood pressure (DBP (continuous), type 2 DM (dichotomous), fasting glucose (dichotomous), total cholesterol (TC) (dichotomous, continuous), high density lipoprotein (HDL) (dichotomous, continuous), low density lipoprotein (LDL) (dichotomous, continuous), and triglycerides (dichotomous, continuous). Discrepancies between the two reviewers during the screening and/or abstraction stage were resolved by consensus or by a third reviewer (SMG).

### Quality assessment

Quality assessment was completed in duplicate by two independent reviewers (LZ, IS) using the Risk of Bias in Non-randomized Studies of Exposure (ROBINS-E) tool for observational studies, and the Cochrane Risk of Bias tool for clinical trials [22, 23]. For observational studies, risk of bias was assessed for each individual outcome reported within a study. For the assessment of bias due to confounding using the ROBINS-E tool, the minimum set of confounders used to assess study quality included: maternal age, prepregnancy BMI, maternal DM, and maternal education. For all studies, risk of bias was assessed for each individual domain. The overall judgement for risk of bias was determined based on the assessment of the individual domains. Disagreements were resolved by consensus or by a third reviewer (SMG).

### Data analysis

Data were pooled across studies using a restricted maximum-likelihood estimator random-effects model for outcomes reported by at least 3 included studies. The total variability due to between-study heterogeneity was assessed using the I^2^ and Tau-squared statistics. Narrative synthesis according to Synthesis Without Meta-analysis (SWiM) reporting guidelines was used to summarize outcomes for which pooling was not feasible due to heterogeneity in outcome definitions or an insufficient number of studies [24]. Publication bias assessed using tests for funnel plot asymmetry were not performed since less than 10 studies were included in the meta-analysis [25]. Statistical analysis was conducted using R version 4.1.2 (R Foundation for Statistical Computing, Vienna, Austria).

## Results

**Fig. 1:**
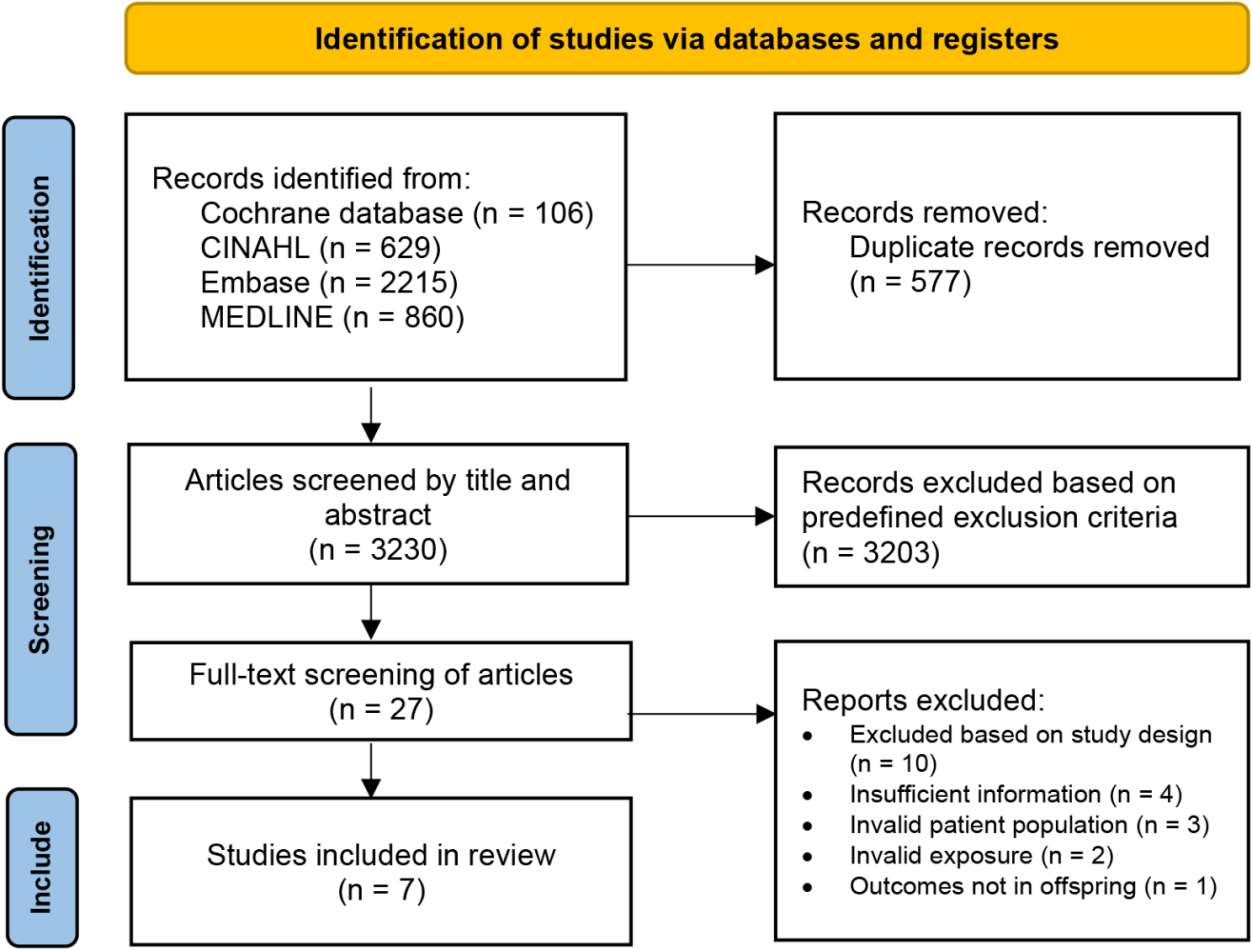
PRISMA flow diagram

The search identified 3221 articles, of which 7 were included (Fig. 1). Characteristics of the included studies are summarized in Table 1. All studies were conducted outside of North America, where six studies were observational and one was a randomized controlled trial. The studies ranged in size from 250 to > 1 million children with a follow-up time of 6 to 20 years. Most studies were conducted using population-based registry data, while one study used data from electronic medical records at a tertiary care center, and four studies used primary data collected through questionnaires at in-person visits [20, 26–29].

**Table 1:** Characteristics of studies examining the effect of maternal hypothyroidism in pregnancy and metabolic outcomes in children

Study	Country	Design	Study Period	Follow-up time	Hypothyroidism definition	Proportion of Women with Hypothyroi-dism (%)	Sample Size	% Female	Outcomes
Godoy 2014 [29]	Netherlands	Prospective cohort	2001 to 2005	6 years from birth	Maternal serum samples obtained in early pregnancy (median = 13.2 weeks); overt hypothyroidism (TSH > 4.04 mIU/L and FT4 < 10.4 pmol/L), subclinical hypothyroidism (TSH > 4.04 mIU/L and FT4 > 10.4 pmol/L)	5.12 (204/3985)	3930	51	BMI; total body and abdominal fat distribution; blood pressure; left ventricular mass
Andersen 2021 [20]	Denmark	Cohort; case-cohort	1997 to 2003	7 years from birth	Cohort 2a: ICD-8 and ICD-10 from register; cohort 2b and 2c: TSH from blood samples in early pregnancy, based on previously established method- and pregnancy week-specific reference ranges within the cohort	Cohort 2a: 1.22 (494/40585) Cohort 2b: 4.94 (210/4255)	39,668	Not reported	BMI; waist circumference
Rytter 2016 [21]	Denmark	Case-cohort	1988 to 1989	19–20 years from birth	Maternal serum samples obtained in week 30 of pregnancy; hypothyroidism defined as TSH > 3 mIU/L and fT4 < 18.8 pmol/L	7.34 (48/654)	654	32	BMI; waist circumference; blood pressure
Heikkinen 2017 [27]	Finland	Population-based cohort	1985 to 1986	16 years from birth	Maternal serum samples obtained in early pregnancy (mean = 10.7 weeks); TSH > 3.1 mU/L in the first trimester and > 3.5 mIU/L in the second trimester, and fT4 < 11.40 pmol/L in first trimester and < 11.09 pmol/L in second trimester	7.56 (375/4957)	4176	48.70	BMI; waist circumference; blood pressure; fasting glucose; lipids and lipoproteins; insulin resistance
Eshkoli 2019 [26]	Israel	Retrospective population-based cohort	1991 to 2014	18 years from birth	Maternal report from midwife and obstetrician, and routine review of all computerized medical records from the hospital and ambulatory setting	1.1	217,910	49.10	Total endocrine morbidity (thyroid disease, diabetes mellitus, hypoglycemia, obesity)
Miao 2021 [8]	Denmark	Population-based cohort	1978 to 1998	18 years from at least 8 years old (oldest participant 38 years)	At least one inpatient diagnosis of hypothyroidism (ICD-8 codes 243–244, and ICD-10 codes E03 and E89.0)	1.1	1,041,448	49.40	Cardiovascular disease (hypertension, ischemic heart disease, arrhythmia, atrial fibrillation, stroke, acute myocardial infarction)
Muller 2020 [28]	United Kingdom	Randomized controlled trial	2002 to 2010	9 years	Maternal blood samples obtained in early pregnancy (median = 12 weeks 3 days); hypothyroidism defined as FT4 < 2.5th percentile and/ or TSH > 97.5th percentile of the cohort	Not reported	248	53	BMI; lean, fat, and bone mass; blood pressure, augmentation indiex, aortic pulse-wave velocity; thyroid function, lipids, insulin and adiponectin

*Abbreviations* TSH, thyroid-stimulating hormone; fT4, free thyroxine; BMI, body mass index

The prevalence of maternal hypothyroidism ranged from 1.1 to 7.6% across studies. The exposure window (prior to or after pregnancy) and method of ascertainment of the exposure (i.e., self-reports, diagnoses, biochemical markers) varied widely across studies (Table 1). Reported outcomes and definitions varied across studies, with several studies using continuous measures (i.e., BMI and SBP) and other studies using dichotomous measures (i.e., hypertension, type 2 diabetes mellitus). A detailed summary of the exposure and outcomes assessed in each study are available in Additional file 3.

Overall, the quality of included studies ranged from low to high risk of bias with the majority of studies (71%) rated as being low or moderate risk of bias. Two studies were rated as being at low risk of bias, three studies at moderate risk of bias, and two studies at high risk of bias [8, 20, 21, 26–29]. The most common reasons for a moderate or high risk of bias rating was due to the methods of accounting for missing data and the use of self-reported outcome measures (Fig. 2a). An overall summary of the risk of bias by outcome is depicted in Fig. 2b.

**Fig. 2a:**
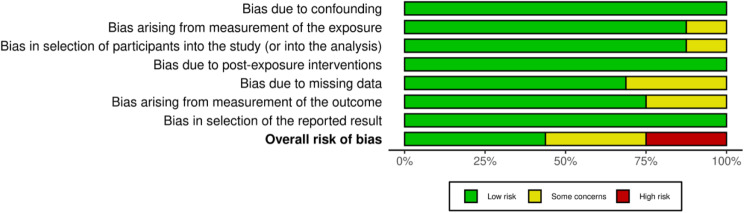
Risk of bias summary by domain

**Fig. 2b:**
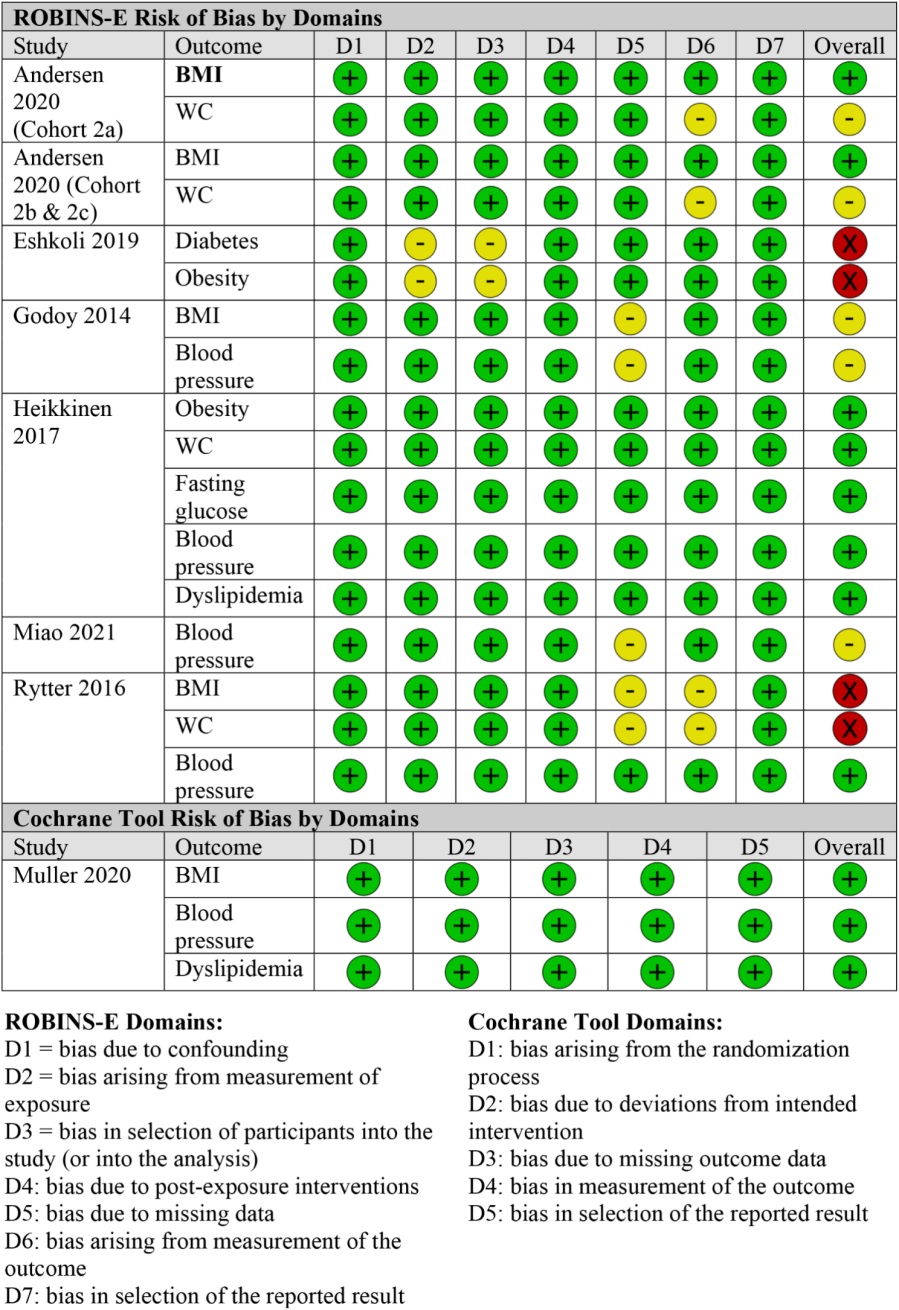
Risk of bias assessment by outcome and tool domain

### Obesity outcomes

BMI was reported in six of the seven included studies [20, 21, 26–29]. The definition and method of reporting varied across studies with 4 studies using continuous BMI, 2 with a categorical or dichotomized definition, and one with both. Three of the seven studies included WC as a proxy measure of obesity, with sex-specific cutoffs (80 cm in girls and 87.5 cm in boys) to define obesity [20, 21, 27]. The overall quality of reporting and ascertainment for this outcome was moderate in the included studies, with a lower quality rating for WC outcomes compared to BMI outcomes due to the use of self-reported WC measures (Fig. 2b).

**Fig. 3:**
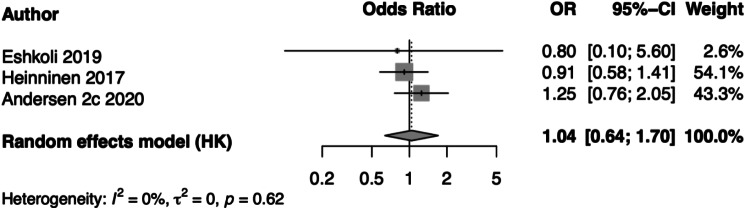
Association of maternal hypothyroidism exposure and BMI-defined obesity

In the seven individual studies, maternal thyroid status was not associated with obesity in offspring. However, the study by Godoy et al. found that higher free thyroxine (fT4) concentrations were inversely associated with childhood BMI [29]. In the pooled analysis of obesity as a dichotomous measure, maternal hypothyroidism was not associated with higher BMI in offspring across 3 studies (OR = 1.04, 95% CI: 0.64, 1.70, I^2^ = 0%, τ^2^ = 0; Fig. 3). Pooling was not performed for WC or categorical measures of obesity due to an insufficient number of studies with similar outcome definitions.

### Hypertension

Hypertension was reported in five of seven included studies, with one study using a recorded diagnosis and four studies using repeat blood pressure measurements. The risk of bias for this outcome was rated as low to moderate across studies due to methods used to handle missing data in two studies (Fig. 2b).

In three studies, maternal hypothyroidism during pregnancy was not associated with elevated blood pressure [21, 27, 29]. However, the study by Miao et al. found that offspring exposed to maternal hypothyroidism were more likely to be diagnosed with hypertension, with a sibling analysis (to account for shared genetic and environmental factors that were not adjusted for in the primary analysis) providing further support for this finding (primary analysis: aHR 1.81 95% CI 1.21, 2.69; sibling analysis: aHR 2.01, 95% 1.20, 3.36; Additional file 3) [8]. In addition, the study by Rytter et al. identified an association between maternal hypothyroidism and increased SBP in offspring (adjusted mean difference 3.6, 95% CI 0.2, 7.0; Additional file 3) [21]. However, no association was found with DBP [19].

### Diabetes mellitus

Type 2 DM was reported in three of the seven included studies, defined by recorded diagnoses and/or biochemical measures of fasting glucose [26–28]. None of these studies reported an association between maternal hypothyroid status and type 2 DM in offspring. However, the point estimate from the Eshkoli study was consistent with an increased risk of diabetes in exposed offspring (Risk Ratio [RR] 2.7, 95% CI 0.7, 10; Additional file 3) [26]. This study also found a higher risk of hypoglycemia in offspring who were exposed to maternal hypothyroidism in pregnancy (RR 2.9, 95% CI 1.4, 6.2; Additional file 3) [26]. However, in the study by Muller et al., where they measured insulin levels, they found no increased risk of abnormal insulin levels associated with exposure to maternal hypothyroidism in pregnancy (euthyroid [*n* = 191]: 4.90 µIU/mL, IQR 3.60–6.90, untreated hypothyroid [*n* = 57]: 4.40 µIU/mL, IQR 3.30–5.7; *p* = 0.385; Additional file 3) [28]. The overall quality of reporting for this outcome was moderate due to the potential bias in exposure ascertainment (i.e., self-reported) in these studies (Fig. 2b).

### Dyslipidemia outcomes

Two of the seven included studies reported triglycerides and total cholesterol levels as continuous measures. These studies found no association between in utero exposure to maternal hypothyroidism and triglyceride and total cholesterol levels [27, 28]. However, one study found an association between elevated HDL concentrations in offspring born to mothers with untreated hypothyroidism compared to mothers treated with levothyroxine for hypothyroidism (euthyroid [*n* = 191]: 1.22 ± 0.27 mmol/L, untreated hypothyroid [*n* = 57]: 1.26 ± 0.29 mmol/L), although this relationship did not persist when comparing offspring born to women with hypothyroidism and euthyroid individuals [28].

The overall quality of reporting for this outcome was rated as low risk of bias as biochemical assessments were used in both studies (Fig. 2b).

## Discussion

This study found no association between exposure to maternal hypothyroidism and subsequent obesity, type 2 DM, hypertension, and dyslipidemia in offspring, although estimates from individual studies suggest a potential increased risk of elevated SBP and dysglycemia in offspring exposed to maternal hypothyroidism. Overall, the inconsistent findings of included studies suggest that further evidence is needed to determine the impact of maternal hypothyroidism in pregnancy and metabolic outcomes in offspring.

While little is known about the biological mechanism through which maternal thyroid hormone may act on metabolic health in offspring, the synthesized findings from this knowledge synthesis align with findings in experimental settings. A study by Santos et al. found that adult rat offspring exposed to induced gestational hypothyroidism had increased mean arterial pressure, SBP, and DBP, which is hypothesized to be due to an increased sympathetic modulation of vessels [16]. In line with our findings on BMI, a previous animal study by Kemkem et al. did not find increased weight gain among mice offspring exposed to maternal hypothyroidism compared to unexposed offspring [17]. However, the study did find that exposed mice were more likely to be glucose-intolerant and insulin-resistant on high-fat diets, suggesting that offspring born to mothers with hypothyroidism may be more vulnerable to metabolic stress [17]. The study by Eshkoli et al. in this review found an increased risk of hypoglycemia in offspring exposed to maternal hypothyroidism, although this finding may be due to concomitant use of insulin in offspring with type 2 DM rather than a result of fetal programming [30]. Given the consistent findings, additional experimental and observational studies are needed to help further elucidate the biological mechanism for the developmental origins of health and disease (DOHaD) of endocrine and metabolic disorders in children.

Quality assessment using the ROBINS-E tool requires setting a minimal set of confounders to assess the extent of confounding bias. Our minimal set was established based on prior evidence but did not include use of levothyroxine given the lack of evidence to support use of levothyroxine for SCH [31, 32]. Levothyroxine treatment in pregnancy is currently recommended in individuals with overt hypothyroidism. However, as decreased fT4 concentrations were not consistently used to define hypothyroid status across studies, the sample populations of the included studies consisted primarily of individuals with SCH, where evidence for the benefit of use of levothyroxine is unclear [33, 34]. Additionally, use of levothyroxine was not reported consistently across the studies included in this review. Only two studies accounted for levothyroxine use, while one study used recorded prescriptions for levothyroxine to define maternal hypothyroidism status [20, 28, 29]. As a result, there is the potential for exposure misclassification for women with restored thyroid hormone levels as a result of treatment with levothyroxine in this study. However, the effect of levothyroxine use in pregnancy on childhood outcomes is unclear. A recent clinical trial found that levothyroxine use in pregnancy did not improve cognitive outcomes in offspring, suggesting that levothyroxine use may not impact future childhood outcomes [35]. Moreover, no study has specifically examined the effect of levothyroxine use on the metabolic health of offspring. Therefore, it is unclear the extent to which the exclusion of the use of levothyroxine during pregnancy in our minimal set of confounders impacts our assessment of confounding bias.

Characteristics of maternal thyroid function were not consistently reported by included studies. Thyroid antibody positivity (TPO-Ab) has been shown to have differential effects on maternal and fetal complications [36]. Information on thyroid peroxidase antibody (TPO-Ab) positivity was only reported in two studies. The study by Heikkinen et al. found that TPO-Ab positive mothers had a greater risk of adiposity and metabolic syndrome [27, 29]. This finding may suggest that metabolic outcomes may differ in offspring exposed to TPO-Ab positive mothers, potentially through an autoimmune mechanism involved in fetal programming that differs by thyroid hormone concentrations [27, 37]. Since thyroid autoimmunity is the leading cause of thyroid disease in women of reproductive age in developed countries, future studies should consider the role of TPO-Ab positivity and its influence on offspring metabolic health [38].

The thresholds to define maternal hypothyroidism based on biochemical assessment varied across the included studies. The 2017 Clinical Practice Guidelines from the American Thyroid Association (ATA) recommend a TSH threshold of > 4.0 mIU/L prior to or during pregnancy or TSH > 2.5 mIU/L in women with positive TPO-Ab status [39]. However, several included studies were completed prior to the release of these guidelines and may have referred to trimester-specific reference ranges from the 2014 European Thyroid Association guidelines to diagnose hypothyroidism (2.5 mIU/l for the first trimester, 3.0 mIU/l for the second trimester, and 3.0-3.5 mIU/l in the third trimester) [21, 27, 29, 32]. Four studies also used fT4 concentrations to classify maternal hypothyroid status, with fT4 thresholds ranging from < 10.4–18.8 pmol/L or fT4 < 2.5th percentile [21, 27–29]. As current guidelines do not recommend universal screening to detect fT4 concentrations, there is a lack of consensus on the appropriate fT4 reference ranges to define hypothyroidism [32, 40]. The variation in observed effect estimates from studies included in this review may in part be explained by the use of different TSH and fT4 reference ranges.

Although the prevalence of hypothyroidism varies by region, hypothyroidism is generally considered to be underdiagnosed in European communities [41, 42]. Large observational studies conducted in Europe and North America have found that hypothyroidism is undiagnosed in 4–7% of the population [38, 42–44]. The prevalence of hypothyroidism varied across included studies based on whether a recorded diagnosis and/or biochemical values were used to define hypothyroidism. In studies using biochemical TSH thresholds, the prevalence of hypothyroidism ranged from 4.9 to 7.6% [20, 21, 27, 29]. This is in contrast to the prevalence of hypothyroidism of 1% in studies that relied solely on a recorded diagnosis of hypothyroidism [8, 26]. In the study by Andersen et al., which used a recorded diagnosis to define hypothyroidism, the prevalence was 1.2% [20]. However, when biochemical TSH measures were used to define exposure in a random sample of the cohort, 4.9% of women were categorized as having hypothyroidism [20]. The large discrepancy in the prevalence of hypothyroidism observed between and within included studies supports the underdiagnosis of hypothyroidism in pregnancy and the need for improved screening efforts in pregnant individuals or those trying to conceive.

Our study has several strengths. First, a comprehensive literature search in five databases was conducted by a trained medical librarian. Additionally, the review followed a pre-specified registered protocol and PRISMA and SWiM reporting guidelines. Second, screening and data extraction were pilot tested and conducted in duplicate by two independent reviewers. Finally, quality assessment was performed using standardized tools by two independent reviewers.

This study has several potential limitations. First, the heterogeneity in outcome definitions limited our ability to pool estimates across studies. Second, the heterogeneity in exposure definitions across studies (based on recorded prescriptions, TSH cut-offs, and recorded diagnoses) further limited our ability to pool effect estimates across studies. Third, the small number of included studies limited the ability to assess publication bias and conduct subgroup analyses to explore potential sources of heterogeneity. Additionally, not all studies consisted exclusively of child or adolescent populations, with one study including individuals aged up to 25 years in the sample population.

## Conclusions

This study found inconsistent evidence to infer an association between maternal hypothyroidism exposure and metabolic outcomes in offspring, though associations with hypertension and glucose dysregulation are possible. Our findings underscore the need for future longitudinal studies investigating the role of maternal hypothyroidism on offspring metabolic health.

### Electronic supplementary material

Below is the link to the electronic supplementary material.

Supplementary Material 1

## Data Availability

This study used the peer-reviewed literature as the data source. Since the study is based on findings from published studies, the dataset(s) supporting the conclusions of this article are included within the article (and its additional files). The dataset(s) supporting the conclusions of this article is(are) available in the Open Science Framework (OSF) repository, [DOI 10.17605/OSF.IO/QG5M4].
