# Supplementary material for: Maternal hypothyroidism and subsequent metabolic outcomes in children: a systematic review and meta-analysis
Source: BMC Pediatr. 2024 Aug 1;24:490. doi: 10.1186/s12887-024-04963-0 (PMC11293156; doi:10.1186/s12887-024-04963-0)
Supplement: Supplementary file 1 — Supplementary Material 1 [file 12887_2024_4963_MOESM1_ESM.docx]

**Supplementary Table 1: PRISMA checklist**

| **Section and Topic** | **Item #** | **Checklist item** | **Location where item is reported** |
| --- | --- | --- | --- |
| **TITLE** | | |  |
| Title | 1 | Identify the report as a systematic review. | Methods |
| **ABSTRACT** | | |  |
| Abstract | 2 | See the PRISMA 2020 for Abstracts checklist. | Abstract |
| **INTRODUCTION** | | |  |
| Rationale | 3 | Describe the rationale for the review in the context of existing knowledge. | Introduction, Paragraphs 1-3 |
| Objectives | 4 | Provide an explicit statement of the objective(s) or question(s) the review addresses. | Paragraph 4 |
| **METHODS** | | |  |
| Eligibility criteria | 5 | Specify the inclusion and exclusion criteria for the review and how studies were grouped for the syntheses. | Methods: study selection |
| Information sources | 6 | Specify all databases, registers, websites, organisations, reference lists and other sources searched or consulted to identify studies. Specify the date when each source was last searched or consulted. | Methods: search strategy |
| Search strategy | 7 | Present the full search strategies for all databases, registers and websites, including any filters and limits used. | Methods, Appendix |
| Selection process | 8 | Specify the methods used to decide whether a study met the inclusion criteria of the review, including how many reviewers screened each record and each report retrieved, whether they worked independently, and if applicable, details of automation tools used in the process. | Methods: study selection |
| Data collection process | 9 | Specify the methods used to collect data from reports, including how many reviewers collected data from each report, whether they worked independently, any processes for obtaining or confirming data from study investigators, and if applicable, details of automation tools used in the process. | Methods: data extraction and outcomes |
| Data items | 10a | List and define all outcomes for which data were sought. Specify whether all results that were compatible with each outcome domain in each study were sought (e.g. for all measures, time points, analyses), and if not, the methods used to decide which results to collect. | Methods: data extraction and outcomes |
|  | 10b | List and define all other variables for which data were sought (e.g. participant and intervention characteristics, funding sources). Describe any assumptions made about any missing or unclear information. | Methods: data extraction and outcomes |
| Study risk of bias assessment | 11 | Specify the methods used to assess risk of bias in the included studies, including details of the tool(s) used, how many reviewers assessed each study and whether they worked independently, and if applicable, details of automation tools used in the process. | Methods: quality assessment |
| Effect measures | 12 | Specify for each outcome the effect measure(s) (e.g. risk ratio, mean difference) used in the synthesis or presentation of results. | Methods: data analysis |
| Synthesis methods | 13a | Describe the processes used to decide which studies were eligible for each synthesis (e.g. tabulating the study intervention characteristics and comparing against the planned groups for each synthesis (item #5)). | Methods: data analysis |
|  | 13b | Describe any methods required to prepare the data for presentation or synthesis, such as handling of missing summary statistics, or data conversions. | Methods: data analysis |
|  | 13c | Describe any methods used to tabulate or visually display results of individual studies and syntheses. | Table 1 |
|  | 13d | Describe any methods used to synthesize results and provide a rationale for the choice(s). If meta-analysis was performed, describe the model(s), method(s) to identify the presence and extent of statistical heterogeneity, and software package(s) used. | Methods: data analysis |
|  | 13e | Describe any methods used to explore possible causes of heterogeneity among study results (e.g. subgroup analysis, meta-regression). | Methods: data analysis |
|  | 13f | Describe any sensitivity analyses conducted to assess robustness of the synthesized results. | NA |
| Reporting bias assessment | 14 | Describe any methods used to assess risk of bias due to missing results in a synthesis (arising from reporting biases). | NA |
| Certainty assessment | 15 | Describe any methods used to assess certainty (or confidence) in the body of evidence for an outcome. | NA |
| **RESULTS** | | |  |
| Study selection | 16a | Describe the results of the search and selection process, from the number of records identified in the search to the number of studies included in the review, ideally using a flow diagram. | Results Figure 1 |
|  | 16b | Cite studies that might appear to meet the inclusion criteria, but which were excluded, and explain why they were excluded. | Appendix |
| Study characteristics | 17 | Cite each included study and present its characteristics. | Appendix |
| Risk of bias in studies | 18 | Present assessments of risk of bias for each included study. | Appendix |
| Results of individual studies | 19 | For all outcomes, present, for each study: (a) summary statistics for each group (where appropriate) and (b) an effect estimate and its precision (e.g. confidence/credible interval), ideally using structured tables or plots. | Results Figure 1, Table 1-2 |
| Results of syntheses | 20a | For each synthesis, briefly summarise the characteristics and risk of bias among contributing studies. | Results |
|  | 20b | Present results of all statistical syntheses conducted. If meta-analysis was done, present for each the summary estimate and its precision (e.g. confidence/credible interval) and measures of statistical heterogeneity. If comparing groups, describe the direction of the effect. | Results Figure 1 |
|  | 20c | Present results of all investigations of possible causes of heterogeneity among study results. | NA |
|  | 20d | Present results of all sensitivity analyses conducted to assess the robustness of the synthesized results. | NA |
| Reporting biases | 21 | Present assessments of risk of bias due to missing results (arising from reporting biases) for each synthesis assessed. | Results Figure 2 |
| Certainty of evidence | 22 | Present assessments of certainty (or confidence) in the body of evidence for each outcome assessed. | NA |
| **DISCUSSION** | | |  |
| Discussion | 23a | Provide a general interpretation of the results in the context of other evidence. | Discussion Paragraph 1-6 |
|  | 23b | Discuss any limitations of the evidence included in the review. | Discussion Paragraph 8 |
|  | 23c | Discuss any limitations of the review processes used. | Discussion Paragraph 8 |
|  | 23d | Discuss implications of the results for practice, policy, and future research. | Discussion Conclusions |
| **OTHER INFORMATION** | | |  |
| Registration and protocol | 24a | Provide registration information for the review, including register name and registration number, or state that the review was not registered. | Methods: study design and registration |
|  | 24b | Indicate where the review protocol can be accessed, or state that a protocol was not prepared. | Methods: study design and registration |
|  | 24c | Describe and explain any amendments to information provided at registration or in the protocol. | NA |
| Support | 25 | Describe sources of financial or non-financial support for the review, and the role of the funders or sponsors in the review. | Funding |
| Competing interests | 26 | Declare any competing interests of review authors. | Declarations of Interest |
| Availability of data, code and other materials | 27 | Report which of the following are publicly available and where they can be found: template data collection forms; data extracted from included studies; data used for all analyses; analytic code; any other materials used in the review. | Data Sharing |

*From:*  Page MJ, McKenzie JE, Bossuyt PM, Boutron I, Hoffmann TC, Mulrow CD, et al. The PRISMA 2020 statement: an updated guideline for reporting systematic reviews. BMJ 2021;372:n71. doi: 10.1136/bmj.n71

For more information, visit: <http://www.prisma-statement.org/>

**Supplementary Table 2: Search Strategy**

(Set 1 and ((set 2 AND (Set 3 or Set 4 or Set 5 or Set 6 or Set 7)))

Set 1 Pre-existing maternal hypothyroidism or hypothyroidism diagnosed in pregnancy

Hypothyroidism/ or *thyroid gland/ or *Endocrinology/st

Or

((hypothyroid* or "Thyroid-Stimulating Hormone Deficien*" or "TSH deficien*") adj4 (pre-exist* or subclinical or syndrom*)).tw,kw,kf

or

thyroid diseases/ or *Euthyroid Sick Syndromes/ or ((thyroid* or euthyroid or "Low T3" or "High T4") and (syndrom* or disease* or illness* or dysfunction*)).tw,kw,kf

Or

(thyroid*).mp

And

Mothers/ or pregnancy/ or in-utero exposure/ or Prenatal Exposure Delayed Effects/ or

(pregnan* or maternal* or mother* or maternal-f$etal or in-utero or prenatal exposure or prenatal effect$ or embryo* or f$etus* or f$etal* or "f$etal programming" or offspring*).tw,kw,kf

Set 2 pediatric set

exp adolescent/ or exp child/ or exp infant/ or *pediatrics/ or young adult/ or (adolescen* or babies or baby or boy? or boyhood or girlhood or child* or girl? or infan* or juvenil* or kid? or minor or minors* or neonat* or neo-nat* or newborn* or new-born* or paediatric* or pediatric* or perinat* or preschool* or puber* or pubescen* or school* or teen* or toddler? or underage? or under-age? or youth* or preteen* or young adult$).ti,ab,kf. or (pediatric* or paediatric* or infan* or child* or adolescen* or young).jn,jw. or (pediatric* or paediatric* or infan* or child* or adolescen* or young).in.

Set 3 cardiometabolic outcomes

"Cardiometabolic Risk Factors"/ or *Cardiovascular Diseases/pc, ep or (cardiometabolic* or cardio-metabolic*).tw,kw,kf

Set 4: Blood pressure

Hypertension/ or *Blood Pressure/ or *Blood Pressure Monitoring, Ambulatory/

Or

(hypertens* or high blood pressure* or elevated blood pressure* or blood pressure monitor*).tw,kw,kf

Set 5 Dyslipidemia

Dyslipidemias/ or (Dyslipid$emia* or Hyperlipid$emia* or Hypercholesterol$emia* or familial hyperlipid$emia or Hyperlipoprotein$emia* or high cholesterol* or Hypertriglycerid$emia* or Hypolipoprotein$emias* or Hypoalphalipoprotein$emias or Hypobetalipoprotein$emia* or "Smith-Lemli-Opitz Syndrome").tw,kw,kf

Set 6 Obesity

*Obesity/ or Pediatric Obesity/ or Overweight/ or Body Mass Index/ or Body Weight/ or adiposity/ or (obese or obesity or overweight* or "high body mass index" or "high BMI" or "high body weight" or "excessive body weight" or "childhood adipos*").ti,ab,kf.

Set 7 Type 2 diabetes

"Diabetes Mellitus, Type 2"/ or (T2DM or "type 2 diabet*" or "type II diabet*" or "Slow-Onset Diabetes Mellitus").tw,kw,kf

or

*Insulin Resistance/ or (insulin adj2 (sensitiv* or resistan*)).tw,kw,kf

**Ovid MEDLINE(R) and Epub Ahead of Print, In-Process, In-Data-Review & Other Non-Indexed Citations and Daily <1946 to May 23, 2023>**

| **#** | **Searches** | **Results** |
| --- | --- | --- |
| 1 | Hypothyroidism/ or *thyroid gland/ or *Endocrinology/st | 68119 |
| 2 | ((hypothyroid* or "Thyroid-Stimulating Hormone Deficien*" or "TSH deficien*") adj4 (pre-exist* or subclinical or syndrom*)).tw,kw,kf. | 5572 |
| 3 | thyroid diseases/ or *Euthyroid Sick Syndromes/ or ((thyroid* or euthyroid or "Low T3" or "High T4") and (syndrom* or disease* or illness* or dysfunction*)).tw,kw,kf. | 81279 |
| 4 | thyroid*.mp. | 252090 |
| 5 | or/1-4 | 263639 |
| 6 | ("32794201" or "24386874" or "26991263").ui. | 3 |
| 7 | Mothers/ or pregnancy/ or in-utero exposure/ or Prenatal Exposure Delayed Effects/ or (pregnan* or maternal* or mother* or maternal-f$etal or in-utero or prenatal exposure or prenatal effect$ or embryo* or f$etus* or f$etal* or "f$etal programming" or offspring*).tw,kw,kf. | 1790482 |
| 8 | 5 and 7 | 20730 |
| 9 | exp adolescent/ or exp child/ or exp infant/ or *pediatrics/ or young adult/ or (adolescen* or babies or baby or boy? or boyhood or girlhood or child* or girl? or infan* or juvenil* or kid? or minor or minors* or neonat* or neo-nat* or newborn* or new-born* or paediatric* or pediatric* or perinat* or preschool* or puber* or pubescen* or school* or teen* or toddler? or underage? or under-age? or youth* or preteen* or young adult$).ti,ab,kf. or (pediatric* or paediatric* or infan* or child* or adolescen* or young).jn,jw. or (pediatric* or paediatric* or infan* or child* or adolescen* or young).in. | 6299452 |
| 10 | "Cardiometabolic Risk Factors"/ or *Cardiovascular Diseases/pc, ep or (cardiometabolic* or cardio-metabolic*).tw,kw,kf. | 57370 |
| 11 | Hypertension/ or *Blood Pressure/ or *Blood Pressure Monitoring, Ambulatory/ | 313645 |
| 12 | (hypertens* or high blood pressure* or elevated blood pressure* or blood pressure monitor*).tw,kw,kf. | 524928 |
| 13 | 11 or 12 | 621014 |
| 14 | Dyslipidemias/ or (Dyslipid$emia* or Hyperlipid$emia* or Hypercholesterol$emia* or familial hyperlipid$emia or Hyperlipoprotein$emia* or high cholesterol* or Hypertriglycerid$emia* or Hypolipoprotein$emias* or Hypoalphalipoprotein$emias or Hypobetalipoprotein$emia* or "Smith-Lemli-Opitz Syndrome").tw,kw,kf. | 112532 |
| 15 | *Obesity/ or Pediatric Obesity/ or Overweight/ or Body Mass Index/ or Body Weight/ or adiposity/ or (obese or obesity or overweight* or "high body mass index" or "high BMI" or "high body weight" or "excessive body weight" or "childhood adipos*").ti,ab,kf. | 656242 |
| 16 | "Diabetes Mellitus, Type 2"/ or (T2DM or "type 2 diabet*" or "type II diabet*" or "Slow-Onset Diabetes Mellitus").tw,kw,kf. | 235232 |
| 17 | *Insulin Resistance/ or (insulin adj2 (sensitiv* or resistan*)).tw,kw,kf. | 123659 |
| 18 | 16 or 17 | 319992 |
| 19 | 10 or 13 or 14 or 15 or 18 | 1494136 |
| 20 | 9 and 19 | 356554 |
| 21 | 8 and 20 | 860 |
| 22 | remove duplicates from 21 | 860 |
| 23 | ("32794201" or "24386874" or "26991263" or "34531830" or "25423569").ui. | 5 |
| 24 | 21 and 23 | 5 |

**Supplemental Table 3. Summary of crude and adjusted effect estimates for cardiometabolic outcomes in offspring exposed to maternal hypothyroidism.**

| **Outcome** | **Godoy** | **Andersen** | **Rytter** | **Heikkinen** | **Eshkoli** | **Miao** | **Muller** |
| --- | --- | --- | --- | --- | --- | --- | --- |
| **Main Results** | Lower maternal TSH levels were associated with lower childhood BMI, total fat mass, abdominal subcutaneous fat mass area and diastolic blood pressure, but not with preperitoneal abdominal fat mass area, systolic blood pressure or left ventricular mass | No association | Offspring of subclinical hypothyroid women had higher systolic blood pressure compared to offspring of euthyroid women. No association was found with offspring BMI, waist circumference, and diastolic blood pressure. | No association | Maternal hypothyroidism appears to be independently associated with long-term pediatric endocrine morbidity of the offspring. | Maternal hypothyroidism is associated with an increased risk of CVD in offspring. | No association |
| **Obesity** | | | | | | | |
| Diagnosis | NR | aRR 1.25 95% CI (-0.76, 2.05) | NR | NR | cRR = 0.8 (0.1-5.6), p = 0.813 | NR | NR |
| BMI | SDS based on hypothyroid diagnosis = -0.07 (-0.21, 0.08)^b^; linear trend based on SD change of TSH = -0.01(-0.04, 0.2)^a^; linear trend based on SD change of fT4 = -0.07 (-0.10, -0.04)^a^, p < 0.05 | aMD in BMI z-score= -0.01 (-0.14, 0.13) | aMD^c^ = 0.49 95% CI (-0.41, 1.39) | aOR = 0.91 (0.58, 1.41) based on BMI ≥ 25 aOR | NR | NR | Unexposed: 17.3 (15.9-19.5); exposed: 17.0 (15.8-19.3); p = 0.587 |
| WC | NR | aMD = 0.24 (-0.49, 0.96) | aMD^c^ = 1.2 95% CI (-1.8, 4.2) | aOR = 0.90 (0.58, 1.39) based on girls ≥ 80cm; boys ≥87.5cm | NR | NR | NR |
| **Diabetes** | | | | | | | |
| Diagnosis | NR | NR | NR | 0.73 (0.48, 1.11) where fasting glucose ≥5.6mmol/L | cRR = 2.7 (0.7-10), p = 0.163 | NR | Unexposed: median = 4.40 μIU/mL (IQR 3.30-5.7), exposed: median = 4.90 μIU/mL (IQR 3.60-6.90); p = 0.385 |
| Hypoglycemia | NR | NR | NR | NR | cRR 2.9, 95% CI 1.4, 6.2 | NR | NR |
| **Hypertension** | | | | | | | |
| Diagnosis | NR | NR | NR | aOR = 1.08 (0.75, 1.57) | NR | aHR = 1.81 (1.21, 2.69) | NR |
| SBP | SDS based on hypothyroid diagnosis = 0.09 (-0.08, 0.25)^b^; linear trend based on SD change of TSH = 0.02(-0.01, 0.05)a; linear trend based on SD change of fT4 = -0.02 (-0.05, 0.02)^a^ | NR | aMD^c^ = 3.6 95% CI (0.2, 7.0) | NR | NR | NR | Unexposed: 124.8 ± 12.9, exposed: 126.9 ± 10.4; p = 0.330 |
| DBP | SDS based on hypothyroid diagnosis = -0.04 (-0.21, 0.13)^b^; linear trend based on SD change of TSH = 0.04 (0.01, 0.08)a, p < 0.05; linear trend based on SD change of fT4 = -0.02 (-0.06, 0.01)^a^ | NR | aMD^c^ = 2.3 95% CI (-0.2, 4.9) | NR | NR | NR | Unexposed: 63.0 ± 7.7, exposed: 61.8 ± 9.0; p = 0.497 |
| **Dyslipidemia** | | | | | | | |
| Triglycerides | NR | NR | NR | aOR = 1 (0.48, 2.08) | NR | NR | Unexposed median = 0.70 (IQR 0.50-1.00), exposed: median = 0.80 (IQR 0.62-0.88), p = 0.789 |
| Total cholesterol | NR | NR | NR | aOR = 0.85 (0.58, 1.26) | NR | NR | Unexposed: 4.35 ± 0.63, exposed: 4.41 ± 0.62; p = 0.398 |
| HDL | NR | NR | NR | aOR = 1.3 (0.83, 2.02) | NR | NR | Unexposed: 1.22 ± 0.27, exposed: 1.26 ± 0.29; p = 0.178 |
| LDL | NR | NR | NR | aOR = 0.94 (0.59, 1.48) | NR | NR | NR |
| **Adjustment** | Minimally adjusted model^a^ = gestational age at intake, child’s sex and age at outcome measurement; fully adjusted model^b^ = gestational age at enrolment, maternal age, ethnicity, educational level, parity, pre-pregnancy body mass index, gestational weight gain, smoking, folic acid supplementation, gender, gestational age at birth, birth weight, breastfeeding, child’s age at measurement and child’s height (fat mass outcomes) or child’s body mass index (cardiovascular outcomes). | Year of child's birth, maternal age, parity, country of birth, geographical residence, educational level, smoking in pregnancy, prepregnancy alcohol intake, prepregnancy BMI and diabetes mellitus. | Model 2^c^: maternal prepregnancy BMI, maternal education, smoking during pregnancy, maternal age, parity, maternal dietary iodine intake, and offspring sex | Maternal age, smoking, parity and overweight/obesity | No adjustments for relative risk; maternal, age, birth weight, preterm birth, induction of labor, mode and delivery adjusted for in hazard ratio of cumulative endocrine morbidity | Birth, sex, maternal parity, maternal age at childbirth, maternal education, and maternal employment. | Age, sex (children analysis only), ethnicity, socioeconomic status, and smoking during pregnancy |

Outcome abbreviations: BMI = body mass index, WC = waist circumference, SBP = systolic blood pressure, DBP = diastolic blood pressure, HDL = high density lipoprotein, LDL = low density lipoprotein.

Categorical outcomes: RR = risk ratio; OR = odds ratio; HR = hazards ratio. Continuous outcomes: SDS = standard deviation scores; MD = mean difference.
